# Protein interference applications in cellular and developmental biology using DARPins that recognize GFP and mCherry

**DOI:** 10.1242/bio.201410041

**Published:** 2014-11-21

**Authors:** Michael Brauchle, Simon Hansen, Emmanuel Caussinus, Anna Lenard, Amanda Ochoa-Espinosa, Oliver Scholz, Simon G. Sprecher, Andreas Plückthun, Markus Affolter

**Affiliations:** 1 Biozentrum, University of Basel, Klingelbergstrasse 50/70, 4056 Basel, Switzerland; 2 Department of Zoology, University of Fribourg, Chemi du Musée 10, 1700 Fribourg, Switzerland; 3 Department of Biochemistry, University of Zurich, Winterthurerstrasse 190, 8057 Zurich, Switzerland

**Keywords:** DARPin, GFP, mCherry, Protein interference

## Abstract

Protein–protein interactions are crucial for cellular homeostasis and play important roles in the dynamic execution of biological processes. While antibodies represent a well-established tool to study protein interactions of extracellular domains and secreted proteins, as well as in fixed and permeabilized cells, they usually cannot be functionally expressed in the cytoplasm of living cells. Non-immunoglobulin protein-binding scaffolds have been identified that also function intracellularly and are now being engineered for synthetic biology applications. Here we used the Designed Ankyrin Repeat Protein (DARPin) scaffold to generate binders to fluorescent proteins and used them to modify biological systems directly at the protein level. DARPins binding to GFP or mCherry were selected by ribosome display. For GFP, binders with K_D_ as low as 160 pM were obtained, while for mCherry the best affinity was 6 nM. We then verified in cell culture their specific binding in a complex cellular environment and found an affinity cut-off in the mid-nanomolar region, above which binding is no longer detectable in the cell. Next, their binding properties were employed to change the localization of the respective fluorescent proteins within cells. Finally, we performed experiments in *Drosophila melanogaster* and *Danio rerio* and utilized these DARPins to either degrade or delocalize fluorescently tagged fusion proteins in developing organisms, and to phenocopy loss-of-function mutations. Specific protein binders can thus be selected *in vitro* and used to reprogram developmental systems *in vivo* directly at the protein level, thereby bypassing some limitations of approaches that function at the DNA or the RNA level.

## INTRODUCTION

Protein–protein interactions are at the basis of virtually all biological phenomena. Therefore, it is of high importance to discover which proteins interact, to understand where and how they form interfaces in the cell and to discover what the biological consequences of their interactions are. Experimental and computational approaches are now underway attempting to map phenotype to genotype down to the residue level (Ryan et al., 2013), but achieving this goal lies still far in the future. Ultimately, protein interactions that are well understood cannot only be inhibited but also used to engineer desired cellular outcomes. Such advances have impact in diverse areas of basic biological research and possibly also in future medical applications.

To study individual protein–protein interactions, it is desirable to specifically manipulate an interaction of interest. To analyze a single protein–protein interaction a very specific approach has to be chosen, since the removal of a protein would result in the cumulative loss of all interactions of that protein. Provided that the protein–protein interaction site is known, this can be achieved by mutating the required amino acid(s), however, this information is rarely available. Another possibility is to use antibodies that can mask a specific epitope on the surface of an extracellular or secreted protein, or, after cell permeabilization, also intracellular proteins. Indeed, it has been shown that an antibody can inhibit only a single function of a multifunctional protein containing an extracellular domain, e.g. by Ingvarsen et al. (Ingvarsen et al., 2013). However, this approach cannot be used within a living cell, since most antibodies cannot be functionally expressed, as they contain disulfides and will generally not fold in the reducing cytoplasm. On the other hand, an engineered protein binder, e.g. a peptide, small molecule or an exogenous protein can successfully interfere with a specific protein–protein interaction also intracellularly. For example, small molecule drugs often interact highly specifically with a particular binding pocket of a protein in order to modify a biological outcome otherwise leading to a disease. However, no specific small molecule is available for the great majority of targets or specificity cannot be ascertained. This situation is even much more dismal for proteins of model organisms, since almost all small molecules are being developed for human targets.

Over the past decade, a number of non-antibody scaffolds have been engineered to identify protein binders that can be used to manipulate biological systems (Binz and Plückthun, 2005). Designed Ankyrin Repeat Protein based binders (DARPins) are one example of such a designed scaffold (Boersma and Plückthun, 2011). They were created through consensus analysis of natural ankyrin repeat proteins and are characterized by low molecular weights (ca. 14–18 kDa), high biophysical stability and efficient production in *E. coli* even when the binding surface is randomized (Binz et al., 2003). Such surface-randomized libraries allow the selection of binders with picomolar to low nanomolar affinities (Binz et al., 2003; Zahnd et al., 2007) and converting the selection technology to high throughput has generated binders for over 170 targets up to now (J. Schaefer and A.P., unpublished). DARPins have been used for many intracellular applications, e.g. as real-time, real-space fluorescent sensors of the conformation of a kinase (Kummer et al., 2012), as very selective intracellular inhibitors of particular kinases (Parizek et al., 2012), caspases (Schweizer et al., 2007) and other proteases (Amstutz et al., 2005; Kawe et al., 2006), or as tubulin polymerization inhibitor (Pecqueur et al., 2012). A DARPin against VEGF-A has been shown to reduce neovascularization in different animal models (Stahl et al., 2013) and in human patients (Souied et al., 2014) and will soon be entering phase III clinical trials.

Besides generating binders to the target itself, it is possible to select binders to a partner that is frequently used as a fusion protein in research, e.g. GFP. A single-domain antibody of 117 amino acids has been identified from immunized llamas (also called nanobody VHH4), which binds with high affinity and selectivity to GFP (Rothbauer et al., 2008). It has been used to purify GFP-tagged proteins (Rothbauer et al., 2008), interfere with or mis-localize GFP fusion proteins (Schornack et al., 2009), degrade fusion proteins (Caussinus et al., 2012) or to help visualize protein–protein interactions in living cells (Herce et al., 2013). Subsequently, additional GFP-binding nanobodies with alternative epitopes have been described (Kirchhofer et al., 2010), which can be combined to assemble protein complexes (Tang et al., 2013).

Despite the prevalent use of fluorescent proteins in different areas of basic research, no protein binders other than to GFP are currently available that can be used to engineer and manipulate cellular systems containing such fluorescent proteins. Clearly, additional protein binders against GFP and especially other fluorescent proteins would increase the possibilities for combinatorial manipulations. Therefore, we set out to generate a set of high-affinity and highly specific DARPins binding to the fluorescent proteins GFP and mCherry. We show that the identified binders recognize their target *in vitro* and in living cells with high selectivity and affinity. We then use these genetically encoded DARPins to modify protein function in different animal models, either by degrading or re-localizing fluorescent proteins in developing organisms. Together these results highlight the DARPin technology as an efficient platform to generate specific protein binders which open up new possibilities for functional analyses of fluorescent proteins in different cellular and developmental systems.

## RESULTS

### Selection and *in vitro* characterization of GFP- and mCherry-binding DARPins

To identify DARPins that specifically bind to fluorescent proteins we performed four rounds of ribosome display using the N2C and N3C libraries (Binz et al., 2003) with GFP and mCherry as target proteins, respectively. For GFP as a target, the enriched pool of DNA was subcloned into an *E. coli* expression vector and crude extracts of 192 single clones were screened against GFP by ELISA. In the GFP binder selection, approximately 70% of the single clones from the N2C (see Materials and Methods) library were hits that resulted in a high, GFP-specific signal, and around 50% of the clones from the N3C library pool scored as positive. All positive clones were also tested for binding to superfolder GFP (sfGFP), an engineered variant with 10 amino acid differences, most of which are on the surface (Pédelacq et al., 2006) (supplementary material Fig. S1). As this version was not used during the selection, only a fraction of binders initially selected against GFP also showed binding to sfGFP, as expected from the high specificity of DARPins. No clone from the N2C library was found to bind sfGFP, but 13 clones of the N3C library did bind to sfGFP as well (data not shown). To examine binding against several GFP variants in more detail, six clones out of the 13 sfGFP binders were chosen for further analysis (3G61, 3G86.1, 3G86.32, 3G124, 3G146 and 3G168; for an explanation of the nomenclature, see Materials and Methods).

Single clones from the selections against mCherry showed fewer positive hits, ca. 10–15% from the N2C and N3C pools, respectively. Five clones from these positive hits were chosen for further analysis (2m22, 2m74, 2m151, 2m172 and 3m160). We then assessed the specificity, oligomeric state, target affinity and epitope overlap of the selected DARPins after performing a single purification step using immobilized metal affinity chromatography (IMAC) (supplementary material Fig. S2).

**Fig. 1.**
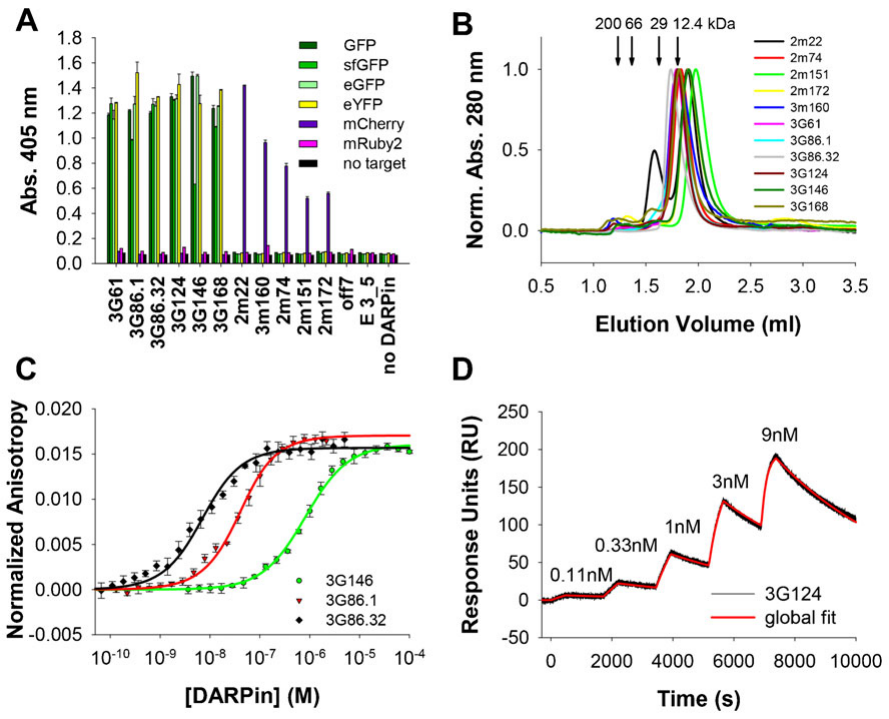
Specificity, oligomeric state and affinity of anti-GFP and anti-mCherry DARPins. (A) ELISA experiments show a high specificity of selected DARPins towards their cognate target and closely related proteins. Off7 is a control DARPin binding to maltose binding protein (MBP), E 3_5 is an unselected DARPin. All bars represent mean values of duplicates, error bars represent standard deviations. (B) Analysis of the oligomeric state by SEC shows that most DARPins are predominantly monomeric. Due to low extinction coefficients at 280 nm for the chromatograms of 2m22 and 2m74 the absorption at 230 nm is shown. Arrows indicate the elution volumes of the molecular weight standard with the respective MWs. (C) Example of FA assays of different DARPins binding to sfGFP. The solid line indicates a fit to a 1:1 binding model. Extracted K_D_s for all DARPins can be found in Table 1. (D) Example of a kinetic titration SPR experiment of 3G124 binding to GFP. The concentrations of the five DARPin injections are indicated in the graph. Fit to a global 1:1 kinetic titration binding model is indicated in red. Extracted association and dissociation rates and K_D_s for several DARPins are summarized in Table 1; additional sensograms are shown in supplementary material Fig. S4.

First, the *in vitro* specificity of the selected DARPins was tested using an ELISA comparing the binding of all selected DARPins, together with control DARPins, to GFP, sfGFP, eGFP, eYFP, mCherry and mRuby2, respectively. Reassuringly, all DARPins showed specific binding to their cognate target (and its close homologs) exclusively (Fig. 1A); no cross-reactivity between GFP- and mCherry-selected DARPins was observed. All anti-GFP DARPins also bound the closely related sfGFP, eGFP and eYFP (Fig. 1A), although sometimes with lower (e.g. 3G146 binding to sfGFP) binding signals. The anti-mCherry DARPins exclusively bound mCherry (Fig. 1A). No DARPin significantly bound mRuby2. Control DARPins off7, selected against maltose binding protein (Binz et al., 2004), and E 3_5, an unselected library member (Binz et al., 2003; Kohl et al., 2003), showed practically no background binding against GFP, sfGFP, eGFP, eYFP, mCherry or mRuby2 and none of the DARPins showed binding above background levels in control wells without coated target (Fig. 1A).

Second, the oligomeric state of all selected DARPins was assessed by analytical size exclusion chromatography (SEC). All but the anti-mCherry DARPin 2m22 showed single elution peaks, in some cases with very small secondary peaks (Fig. 1B). The major DARPin peaks generally eluted with a slight retardation, giving a slightly lower apparent MW than expected (Table 1), calculated using a protein standard consisting of four globular proteins. This indicates that they are exclusively monomeric and compactly folded, but may have a slight interaction with the size exclusion column. Anti-mCherry DARPin 2m22 showed two elution peaks, a main peak at an apparent MW of 9.0 kDa corresponding to the monomeric fraction which is retarded on the column and a minor peak at 32.3 kDa with about half the height, probably corresponding to a dimeric fraction (Fig. 1B; Table 1). Importantly, upon incubation of DARPin 2m22 with an equimolar amount of its cognate target mCherry, all of the mCherry shifts to a single peak of higher molecular weight in SEC, indicating that also the dimeric fraction of 2m22 is incorporated into a 1:1 complex with mCherry (supplementary material Fig. S3).

**Table 1.**
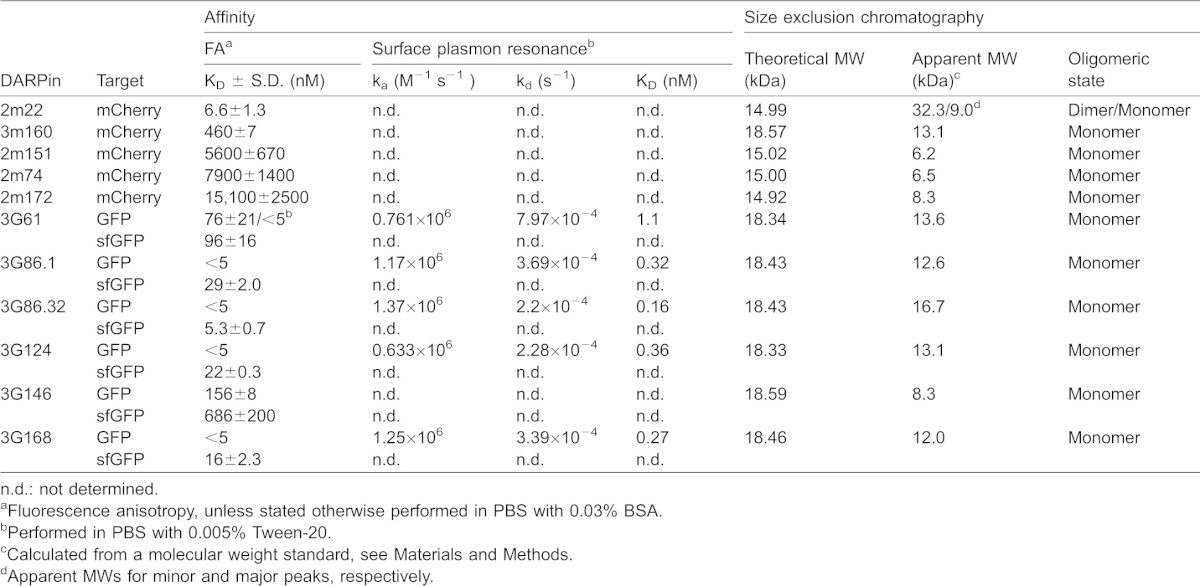
Affinities and oligomeric states of GFP and mCherry-binding DARPins

Third, we measured the affinity of the DARPins for their target by both fluorescence anisotropy (FA) (Fig. 1C; Table 1) and surface plasmon resonance (SPR) (Fig. 1D; Table 1; supplementary material Fig. S4). FA is a very useful method for accurate determination of affinities down to approximately 5 nM, such as those obtained with the non-cognate target sfGFP (Table 1). However, it is not sensitive enough to determine picomolar affinities, since the interaction partners need to be diluted below the dissociation constants (K_D_), resulting in signals which are too weak for precise measurements. Thus, the K_D_ of DARPins 3G61, 3G86.1, 3G86.32, 3G124 and 3G168 were determined with SPR (Fig. 1D; supplementary material Fig. S4), with the best binder, 3G86.32, yielding a K_D_ of 160 pM for its cognate target GFP (Table 1).

In addition, GFP binder 3G61 was measured with both FA and SPR, with the aim to be able to compare the two methods with each other. When measured in the same buffer, we could ascertain that the methods give similar K_D_ values, even though the FA-measured value could not be fitted exactly, as explained above (Table 1). During these measurements, we observed that the K_D_ for this particular DARPin appears to depend on buffer composition (PBS containing either 0.03% BSA (standard buffer for FA) or 0.005% of detergent). Three different detergents, Tween-20, Triton X-100 and decyl-maltoside (DM), were tested and they all increased the affinity of 3G61 to GFP, although to different extents (supplementary material Fig. S5A). In the SPR experiments PBS with 0.005% Tween-20 was used. An increase of affinity induced by detergent was also observed for 3G146 but not for any of the other anti-GFP DARPins (data not shown); the exact molecular cause will require further investigations. We must of course expect that the intracellular situation will be reflected by the absence of detergent-like molecules.

The five selected mCherry binding DARPins had lower affinities, only DARPin 2m22 had a K_D_ in the low nanomolar range; 3m160 had an intermediate affinity with a K_D_ of about 460 nM, while the other mCherry binders had affinities in the µM range (Table 1).

Finally, we performed epitope binning experiments to determine whether the selected DARPins bind to overlapping or to different epitopes on their respective target protein. For the GFP binders, SPR as well as FA measurements were used. In the SPR experiments, two consecutive injections of different anti-GFP DARPins at concentrations saturating all immobilized ligand were made. At the time of the second injection, a large amount of the previously injected anti-GFP DARPin was still bound to the GFP-coated surface. None of the second injections resulted in a signal significantly above the plateau reached with the first injection, suggesting that all anti-GFP DARPins bind to overlapping GFP epitopes (supplementary material Fig. S6). This result was corroborated by the FA experiments, in which GFP was incubated either with no, with one or with two DARPins. Incubation with one anti-GFP DARPin resulted in a significant rise of the anisotropy compared to GFP alone. Addition of a second anti-GFP DARPin, however, did not increase the anisotropy further in any case tested (supplementary material Fig. S7). Epitope binning using FA was also attempted for the mCherry binders, but the results were inconclusive; this is likely due to the different MWs of the anti-mCherry DARPins resulting from the use of different libraries and/or the significantly different K_D_ values found among the mCherry binders. In conclusion, we identified here mainly monomeric DARPin binders for GFP and mCherry that specifically bind to their target *in vitro* with high affinities (Fig. 1; Table 1).

### Anti-GFP and anti-mCherry DARPins bind the respective fluorescent protein in cultured cells

**Fig. 2.**
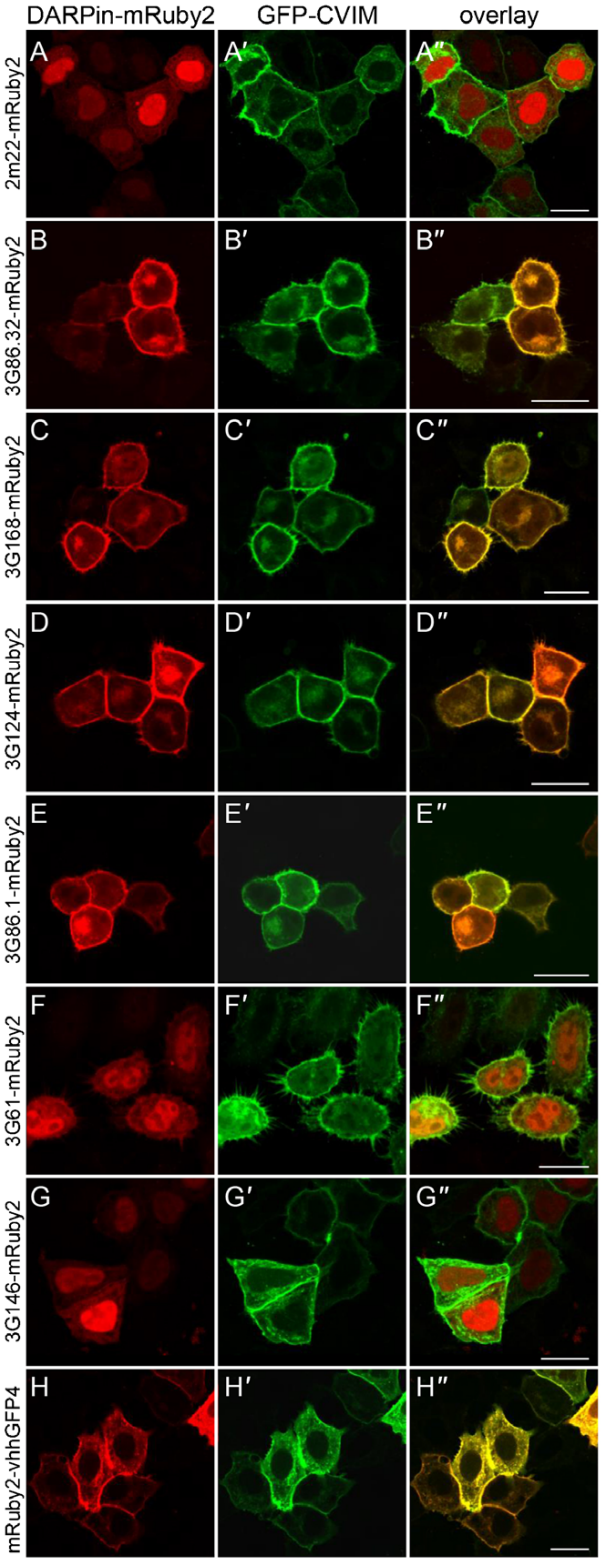
Binding of anti-GFP-DARPin-Ruby2 fusions to GFP in HeLa cells. Shown are HeLa cells transiently overexpressing a DARPin-mRuby2 fusion protein (A–G) (H is an mRuby2-nanobody fusion) together with a GFP version tethered to the plasma membrane (GFP-CVIM, A′–H′). Overlap of fluorescent signal indicates binding of the respective anti-GFP-DARPin-mRuby2 fusion to GFP-CVIM, indicated by the yellow fluorescent signal at the plasma membrane. (A–A″) As expected, the anti-mCherry DARPin 2m22-mRuby2 fusion protein, which does not recognize GFP, is not recruited to the plasma membrane. (B–B″) 3G86.32-mRuby2, (C–C″) 3G168-mRuby2, (D–D″) 3G124-mRuby2 as well as (E–E″) 3G86.1-mRuby2 fusion proteins localize to the plasma membrane where they interact with GFP-CVIM. On the other hand, low affinity (F–F″) 3G61-mRuby2 and (G–G″) 3G146-mRuby2 fusion proteins localize to the cytoplasm and nucleus, indicating that they cannot interact sufficiently with GFP-CVIM anchored in the plasma membrane. (H–H″) Positive control mRuby2-VHH-GFP4 fusion protein localizes to the plasma membrane. Unprimed letters, mRuby2 channel; primed letters, GFP channel; double primed letters, overlay. Scale bars are 20 µm.

Next we wanted to test the intracellular expression and binding behavior of the identified DARPins in a complex cellular environment. For this purpose, we fused the anti-GFP DARPins to yet another orthogonal fluorescent protein, mRuby2, to which they have no affinity as verified by ELISA (Fig. 1A), which allowed us to directly visualize the subcellular location of the DARPin fusion proteins within the cell (Fig. 2). Since the DARPins fold well even as fusion proteins to fluorescent proteins, unlike antibody scFv fragments (Griep et al., 1999), such DARPin-GFP fusion proteins have been routinely used, e.g. when testing DARPins for binding to surface receptors by FACS (Stefan et al., 2011). Upon transfection of individual DARPin-mRuby2 (or -GFP) fusion constructs into HeLa cells, we observed a diffuse cytoplasmic and enriched nuclear fluorescent signal (for an example, see Fig. 2A). This localization was the same as for the respective fluorescent protein itself without a fused DARPin (supplementary material Fig. S8A).

We then co-expressed these DARPin-mRuby2 fusions with membrane-bound versions of the fluorescent proteins they recognize; for this purpose, a “CaaX” motif (Kitten and Nigg, 1991), here in the form of CVIM, was added to the C-termini of GFP or mCherry, which leads to farnesylation of the fluorescent proteins (Fig. 2A′). We predicted that the DARPin-fusions would change their subcellular location upon binding to their respective fluorescent target, which is anchored in the plasma membrane.

**Fig. 3.**
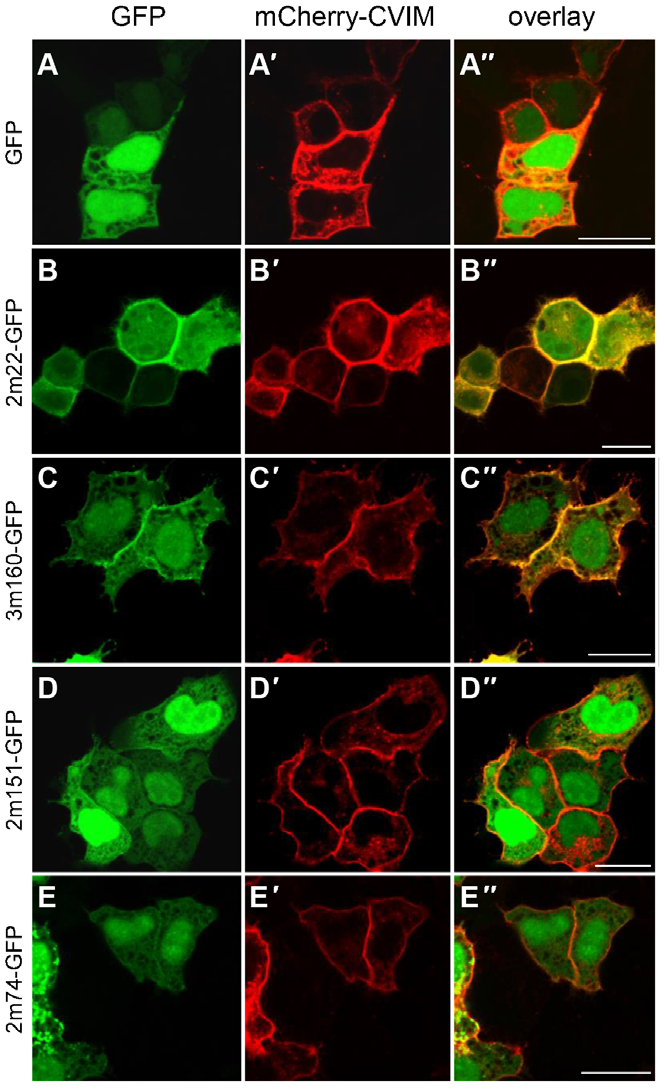
Binding of anti-mCherry-Darpin-GFP fusions to mCherry in HeLa cells. Shown are HeLa cells transiently overexpressing GFP (A) or anti-mCherry-GFP fusion proteins (B–E) together with a mCherry version tethered to the plasma membrane (mCherry-CVIM, A′–E′). Overlap of fluorescent signal indicates binding of the respective anti-mCherry-DARPin-GFP fusion to mCherry-CVIM, indicated by the yellow fluorescent signal at the plasma membrane. (A–A″) As expected, the untethered GFP control does not change its subcellular localization upon co-expression with mCherry-CVIM and remains cytoplasmic and nuclear. (B–B″) 2m22-GFP re-localizes to the plasma membrane where it binds to mCherry-CVIM. (C–C″) The 3m160-GFP fusion protein results in some fluorescent signal at the plasma membrane indicating the weaker affinity to mCherry-CVIM. (D–D″) 2m151-GFP and (E–E″) 2m74-GFP fusion proteins are not significantly recruited to the plasma because of their only micromolar affinity. Unprimed letters, GFP channel; primed letters, mCherry channel; double primed letters, overlay. Scale bars are 20 µm.

We overexpressed six anti-GFP DARPins fused to mRuby2 and assessed their localization upon co-expression with GFP-CVIM (Fig. 2B–H). Indeed, 3G86.32-mRuby2 (Fig. 2B), 3G168-mRuby2 (Fig. 2C), 3G124-mRuby2 (Fig. 2D) and 3G86.1-mRuby2 (Fig. 2E) re-localized to the plasma membrane in the presence of GFP-CVIM, indicating a strong intracellular interaction between these anti-GFP DARPins and GFP-CVIM, consistent with their very high affinity (Table 1). On the other hand, 3G61-mRuby2 (Fig. 2F) and 3G146-mRuby2 (Fig. 2G) failed to re-localize to the plasma membrane. A possible explanation is that their mid-nanomolar affinity as determined by FA (Table 1) is not strong enough to support an intracellular re-localization. To exclude possible steric interference of the plasma membrane with formation of the DARPin-GFP complex (as the CVIM anchor might bring the GFP epitope too close to the membrane), we reversed the experiment by anchoring the tight binding DARPin 3G86.32-mRuby2-CVIM to the plasma membrane and tested if it can recruit cytoplasmic GFP. Indeed, GFP was recruited to the plasma membrane by this DARPin (supplementary material Fig. S8B′), while the lower affinity DARPins, 3G61-mRuby2-CVIM (supplementary material Fig. S8C′) and 3G146-mRuby2-CVIM (supplementary material Fig. S8D′), did not maintain GFP at the plasma membrane, again suggesting that the insufficient affinity is the cause of not allowing a prolonged protein–protein interaction in a cellular environment.

We then repeated this experimental approach to validate a subset of our anti-mCherry DARPins (Fig. 3). Four anti-mCherry DARPins were fused to GFP (which they do not recognize, Fig. 1A) and their localization was tested upon co-expression with a membrane-bound form of mCherry, mCherry-CVIM (Fig. 3A′). We observed that 2m22-GFP (Fig. 3B) and 3m160-GFP (Fig. 3C), which have nanomolar affinities, displayed a membrane-associated localization, indicative of binding to mCherry-CVIM, while 2m151-GFP (Fig. 3D) and 2m74-GFP (Fig. 3E) with micromolar affinities did not change their localization. Furthermore, we also verified that the anti-GFP and anti-mCherry DARPins as Ruby2-fusion proteins only bound to their cognate fluorescent protein target; this was indeed the case as the anti-mCherry-Ruby2 fusion does not bind GFP (Fig. 2A and data not shown).

As a further control, we tested the anti-GFP-nanobody VHH-GFP4 (Rothbauer et al., 2008), fused to mRuby2, and co-expressed it with a GFP-CVIM (Fig. 2H). Indeed, the mRuby2-VHH-GFP4 fusion protein localized to the plasma membrane (Fig. 2H), co-localizing with GFP-CVIM in that location (Fig. 2H′,H″), hereby validating this approach.

Taken together, we found that in a cellular system, only those DARPins which show at least mid-nanomolar affinity in PBS bind to their target. The affinity cut-off between GFP binders and mCherry binders is not identical, since geometric factors may well play a role. In summary, we identified four anti-GFP DARPins (3G86.32, 3G168, 3G124 and 3G86.1), as well as two anti-mCherry DARPins (2m22 and 3m160), which specifically bind to their respective fluorescent protein both *in vitro* and in cultured cells.

### Anti-GFP and anti-mCherry DARPins can be functionalized for *in vivo* experiments in *Drosophila* and zebrafish

**Fig. 4.**
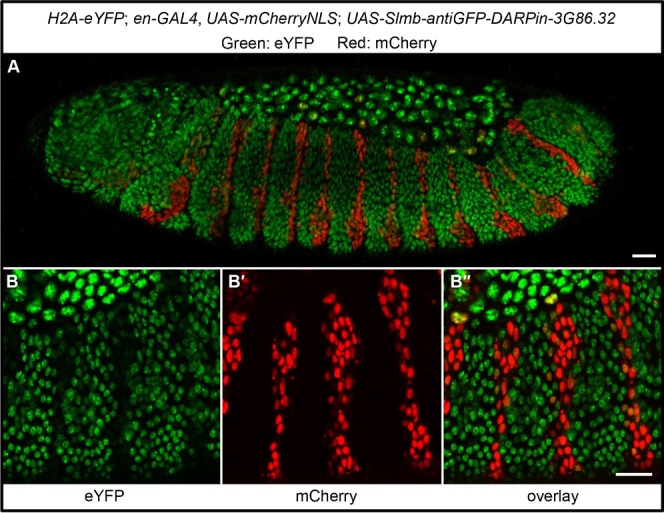
Expression of a Slmb-anti-GFP-DARPin fusion in *Drosophila* embryos degrades eYFP. (A) Shown is an embryo with the following genotype: *H2A-eYFP; en-GAL4, UAS-mCherryNLS; UAS-Slmb-3G86.32*. Nuclei that express the Slmb-anti-GFP-DARPin in the *engrailed* pattern are also expressing unfused mCherry. It can be seen that red cells lost the eYFP signal. (B–B″) Close-up of another embryo showing the channel-specific signal of eYFP (B), mCherry (B′) and the overlay (B″). Note again that cells expressing mCherry have strongly reduced H2A-eYFP signal, indicating efficient eYFP degradation due to the expression of a Slmb-anti-GFP-Darpin. Scale bars are 20 µm.

These successful cell culture experiments encouraged us to test these novel DARPin reagents *in vivo* in multicellular organisms and to evaluate whether the expression of such DARPin fusion constructs would have any deleterious effects in a living organism in the absence of the target fluorescent protein. It has previously been shown that a fusion of the VHH-GFP4 nanobody with a F-box protein can lead to the degradation of GFP-tagged proteins (Caussinus et al., 2012). Using the same experimental approach, we fused the anti-GFP DARPin 3G86.32 to the identical *Drosophila* Slmb F-box domain. We generated transgenic flies expressing the Slmb-3G86.32 fusion protein under a UAS promoter, and drove its expression using the *engrailed* (*en*)-Gal4 driver; at the same time we labelled these cells with mCherry using the same driver. This is expected to lead to an expression of both the DARPin-F-box fusion protein and the (unfused) mCherry in an *engrailed* pattern. We then crossed these flies with a fly strain, in which all cell nuclei express a histone fusion, H2A-eYFP (note that eYFP is also recognized by the anti-GFP DARPins, as shown by ELISA (Fig. 1A)). At the developmental time when the *en* driver becomes active in a stripe-like manner at the posterior part of each body parasegment, the Slmb-3G86.32 fusion protein is expressed in these stripes. The cells within these stripes loose H2A-eYFP (Fig. 4), indicating that the anti-GFP DARPin-F-box fusion protein targeted H2A-eYFP for degradation. These cells can be recognized by their expression of the unfused mCherry. As a control, we also expressed the Slmb-3G86.32 fusion protein under the control of the ubiquitous driver *tub*-Gal4 in a wild-type fly not expressing any GFP fusion protein (data not shown). Such transgenic flies were viable and developed normally, indicating that the presence of the Slmb-3G86.32 fusion protein does not lead to the degradation of any other essential protein, further indicating the high degree of specificity of the anti-GFP DARPin to its respective GFP or eYFP target.

**Fig. 5.**
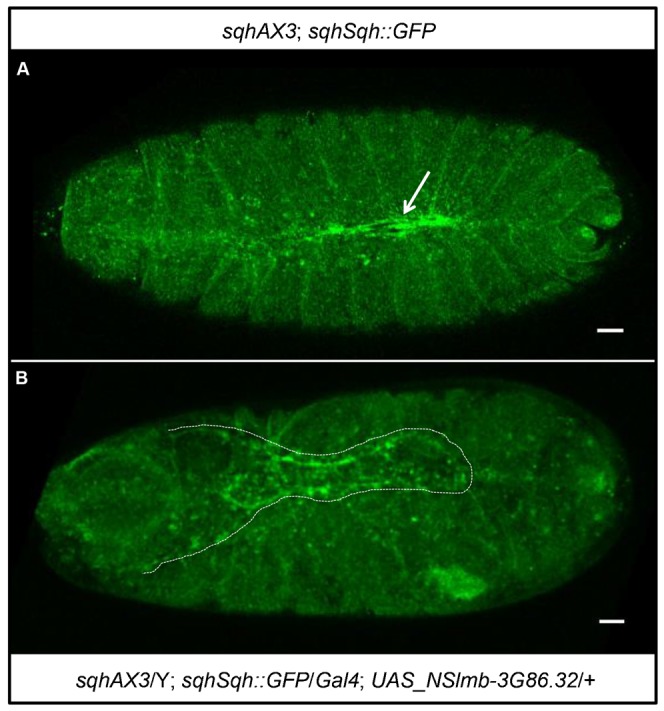
Tissue specific expression of a Slmb-anti-GFP-DARPin fusion in *Drosophila* phenocopies a non-muscle myosin II mutant phenotype. (A) Shown is an embryo with the following genotype: *sqhAX3*; *sqhSqh::GFP*. The arrow points to the normal dorsal closure. (B) Shown is an embryo with the following genotype: *sqhAX3*/Y; *sqhSqh::GFP*/*Gal4*; *NSlmb-3G86.32*/+. The dotted line outlines the “dorsal open” phenotype exposing the amnioserosa. Scale bars are 20 µm.

Next, we attempted to degrade an endogenously GFP-tagged protein in *Drosophila* in order to assess whether we were able to phenocopy the mutant phenotype of the gene of interest. For this purpose, we used a fly line expressing Spaghetti squash (Sqh) (a subunit of the non-muscle myosin II complex) as a GFP fusion protein under the control of its own promoter, hereafter called *sqhSqh::GFP*, thereby rescuing the *sqh* mutant (Fig. 5A) (Royou et al., 2004; also see Caussinus et al., 2012). In such embryos, we expressed the F-Box anti-GFP DARPin fusion protein, Slmb-3G86.32, using an amnioserosa-specific GAL4 driver. These flies exhibited a clear phenotype where the lateral epidermis could not close at the dorsal side, similar to what is observed in strong *sqh* alleles (Young et al., 1993), indicating that the sqhSqh::GFP protein was successfully degraded in the amnioserosa (Fig. 5B). Therefore, mutant phenotypes can be phenocopied using DARPin based reagents.

**Fig. 6.**
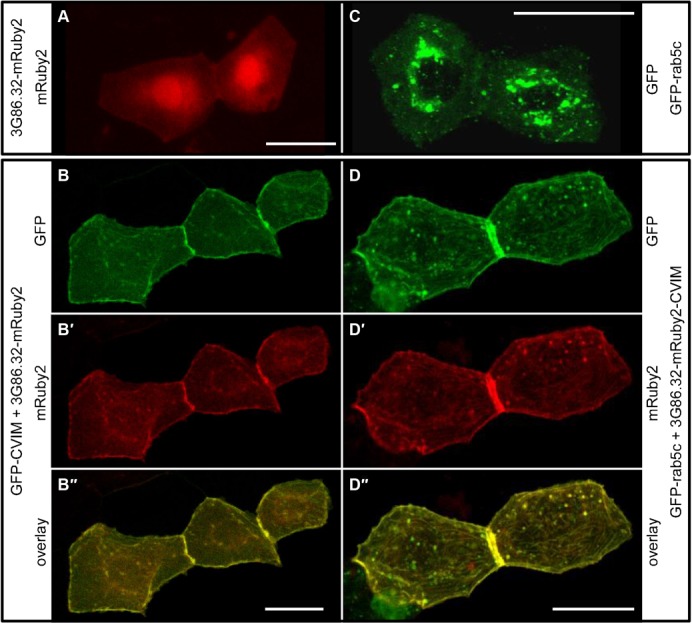
anti-GFP-DARPin fusion proteins can relocalize fluorescent fusion proteins in *D. rerio* embryos. (A,B) 3G86.2-mRuby2 binds GFP in living zebrafish embryos. (A) Control embryo showing the localization of 3G86.32-mRuby2 in two adjacent skin cells. (B–B″) In a zebrafish embryo co-expressing membrane-bound GFP-CVIM (B), 3G86.32-mRuby2 now localizes to the plasma membrane (B′) and shows a virtually complete co-localization with GFP-CVIM (B″). (C,D) membrane-anchored 3G86.32-mRuby2-CVIM recruits GFP-rab5c in living zebrafish embryos. (C) Control embryo showing the localization of GFP-rab5c in two adjacent skin cells. (D–D″) In a zebrafish embryo co-expressing membrane-bound 3G86.32-mRuby2-CVIM (D′), GFP-Rab5C also localizes to the plasma membrane (D) and shows a virtually complete co-localization with 3G86.32-mRuby2-CVIM (D″). Scale bars are 20 µm.

Since zebrafish is an interesting model system for vertebrate development and for live imaging analyses, we also wanted to test an anti-GFP DARPin in living zebrafish embryos. Again, we fused the anti-GFP DARPin 3G86.32 to mRuby2 in order to be able to follow its intracellular localization upon expression, and injected the plasmid encoding this fusion protein into fertilized zebrafish eggs. As expected, we observed mosaic expression (Stuart et al., 1990), and 3G86.32-mRuby2 was detected in various cell types, including skin cells, where we again observed a diffuse cytoplasmic localization together with a nuclear enrichment (Fig. 6A). We next co-injected the 3G86.32-mRuby2 encoding plasmid with a second plasmid encoding GFP-CVIM. Upon co-expression, a dramatic re-localization of 3G86.32-mRuby2 was observed: in skin cells labelled with membrane bound GFP-CVIM, 3G86.32-mRuby2 also localized to the plasma membrane (Fig. 6B–B″). Comparing the different fluorescent channels with each other revealed that the co-localization was virtually complete upon GFP-CVIM expression (Fig. 6B″).

We then wanted to know whether the expression of a localized anti-GFP DARPin can be used to re-localize a functional GFP fusion protein. To test this, we chose GFP-Rab5c, which is normally distributed in a dotted pattern reminiscent of early endosomes (Clark et al., 2011), as this protein, which is involved in vesicle docking and fusion, is attached to the endosomal membrane from the cytoplasmic side. Transient expression of this fusion protein resulted in mosaic zebrafish skin cells with the expected fluorescent distribution of highly dynamic punctate staining in the cytoplasm (Fig. 6C). Upon co-expression of GFP-Rab5c with a membrane anchored 3G86.32-mRuby2-CVIM, we observed that GFP-Rab5c now co-localized with the anti-GFP DARPin to the plasma membrane (Fig. 6D–D″). This result shows that the localization of a GFP-tagged protein in cells of a living organism can be altered by taking advantage of a protein–protein interaction engineered in the lab.

## DISCUSSION

Here we have generated and characterized protein binders selective for either GFP (and closely related fluorescent proteins) or mCherry using the DARPin technology. Importantly, the whole selection was done *in vitro* and therefore differs from the previously identified GFP binders of the nanobody category, which required immunization of a camelid (Rothbauer et al., 2008; Twair et al., 2014). To our knowledge, this is the first time that protein binders to a fluorescent protein other than GFP have been made, now allowing approaches using multiple fluorescent targets at the same time. We went on to show that these binders recognize the respective fluorescent protein in cultured cells, as well as in cells of *Drosophila* and zebrafish embryos, and that the binders can be functionalized in order to modify and control protein function. This is possible since DARPins, which have no cysteines, express well and fold properly in the cytoplasm of any cell. Also, their robust architecture allows them to fold as fusions to many proteins, including fluorescent proteins.

Four of the six GFP-binding DARPins (3G86.1, 3G86.32, 3G124 and 3G168) had the same residues at some variable positions (supplementary material Fig. S9; Table S1); they probably bind the same epitope, as they compete with each other for binding and have similar binding kinetics. These four binders possess a cysteine residue at one of the semi-variable positions, which is not intended in the DARPin design. This residue is at the back of the molecule (away from the binding site) where in the original library (Binz et al., 2003) three different residues (Asn, His or Tyr) are encoded (supplementary material Fig. S9). Indeed, in 3G124 this cysteine was exchanged with a tyrosine without affecting its affinity (data not shown). No sign of aggregation was observed in these proteins despite not using reducing agents in any of the purification steps; in the reducing cytoplasm, this is not an issue.

The structures and epitopes of 3G124 and 3G61 have been determined by protein crystallography (Batyuk et al., manuscript in preparation) and were shown to be different but overlapping, thereby explaining that they compete with each other for binding. 3G61 and 3G146 showed an improved binding affinity to GFP in detergent-containing buffers (supplementary material Fig. S5A). This has nothing to do with an altered oligomeric state of GFP or the DARPin through detergent addition, since 3G61 shows the same behavior towards sfGFP (supplementary material Fig. S5B), which is predominately monomeric in PBS (data not shown) and less aggregation-prone (Pédelacq et al., 2006). Both DARPins show predominantly monomeric behavior in SEC, performed without detergents in the buffer. We believe that 3G61 and 3G146 are to some extent adsorbed by the microtiter plates during the FA assays, which lowers the free DARPin concentration and therefore results in higher apparent K_D_s, but the molecular details will require further investigations. None of the other DARPins show this behavior.

The DARPins selected to bind to GFP also bind to the closely related sfGFP, eGFP and eYFP (96, 99 and 97% sequence identity, respectively; Fig. 1A and supplementary material Fig. S1), albeit with reduced affinity to sfGFP (Table 1), expanding their range of uses. Importantly, none of the DARPins selected against GFP bind mCherry or mRuby2, and vice-versa. This is not surprising, given that the sequences of mCherry and mRuby2 have only 27 and 30% sequence identity to GFP, respectively. Moreover, DARPins selected against mCherry do not bind mRuby2, as these two proteins share only 54% of their sequence (Fig. 1A; supplementary material Fig. S1). As one would expect, the *in vitro* data corresponded well with the observations in cell culture, that is, that the strongest *in vitro* binders also bound their respective fluorescent protein inside cells significantly better than some of the weaker *in vitro* binders.

DARPins can be generated in vitro, without using any immunization, against any target with high affinity and specificity (Boersma and Plückthun, 2011), thus opening up many possibilities for targeting the cellular proteins directly, not only via the detour of targeting an artificial fusion protein as a “recognition tag”. The newly developed LoopDARPins (Schilling et al., 2014) have shown that the rapid selection of specific binders to individual family members of related proteins is possible, thereby allowing to selectively interfere with or manipulate single family members and investigate the consequences in cultured cells or in vertebrate animals; such selections have now been moved to high-throughput technologies (J. Schaefer and A.P., unpublished). Similar approaches have also been used in cell culture to interfere specifically with functions of only particular members from large families of similar proteins (Amstutz et al., 2005; Kawe et al., 2006; Parizek et al., 2012; Schweizer et al., 2007), act as a temporal and spatial sensor of protein conformation and function (Kummer et al., 2012), or to interfere specifically with a subset of protein–protein interactions of a given protein (Yeh et al., 2013).

Here we have shown that the GFP-DARPin can be used in protein degradation assays (Fig. 4), in assays similar to the ones previously used with the GFP nanobody (Caussinus et al., 2012). We believe that none of the two binders is a priori a superior choice in such a degradation experiment which depends to a large extent on, e.g. accessibility of the binder to the fusion protein and/or amino acid sequence and properties of the target. Therefore a quantitative comparison between DARPin and nanobody based protein degradations is meaningless. Indeed, a broad panel of GFP-fusion proteins is being tested in our and other labs; we have previously observed that not all GFP-fusion proteins are easily accessible for degradation using the GFP nanobody (also see Caussinus et al., 2013 for a detailed discussion) and we expect the same with the anti-GFP DARPins. More importantly, however, the DARPin selection procedure is a fully streamlined in vitro approach and specific binders can be selected against new targets in a matter of weeks. In any case, having additional highly specific GFP and mCherry binders at hand now should increase the options of designing experiments, e.g. for protein degradation.

We expect that the DARPins described here will provide useful tools in different fields of biology. Having binders to *different* fluorescent proteins in hand will certainly allow additional biological approaches to be developed and increase their modularity. For example, a bipartite DARPin molecule can easily be engineered in many desired geometries and stoichiometries (e.g. Stefan et al., 2011), thereby bringing GFP- and mCherry-fusion molecules into close proximity in a predetermined orientation, which would allow the assembly of novel protein complexes with predetermined geometry.

These DARPins can be used in any system where GFP or mCherry can be expressed. Indeed, we show for the first time that we can use them in living zebrafish embryos and change the intracellular location of a GFP-tagged protein in that system. We are convinced that such protein binders can be used in virtually all biological systems. Together with new genome editing tools, including TALEN or CRISPR/Cas9 (Hsu et al., 2014), one can envision to influence the location and thus signaling of proteins also in non-classical model systems. The genome of such systems could, for example, be engineered to express specific GFP fusion proteins and these could later be observed in real time, in the absence and the presence of protein binders that re-localize or degrade these fusion proteins.

## MATERIALS AND METHODS

All animal experiments conform to the relevant institutional and regulatory standards of the Swiss Academy of Sciences.

### Ribosome display selection of GFP- and mCherry-binding DARPins

DARPin N3C and N2C libraries (Binz et al., 2004) were used and ribosome display (RD) selections were performed as described (Dreier and Plückthun, 2011). Green Fluorescent Protein (GFP) or mCherry, containing a C-terminal His-tag and an N-terminal avi-tag for *in-vivo* biotinylation during expression, were used as targets. Four rounds of RD with increasing stringency were carried out in solution with pull-down of the ternary complexes using streptavidin-coated magnetic beads. In the fourth round an off-rate selection with 300-fold excess of unbiotinylated GFP or mCherry over biotinylated target was performed. After the fourth round the enriched DNA pools were subcloned into the expression vector pQIq (Simon et al., 2012). For each selection, 192 single clones of each pool were screened by crude extract ELISA as described previously (Binz et al., 2004; Zahnd et al., 2006). All single clones were also screened for binding to superfolder GFP (sfGFP). The nomenclature of the binders is as follows: the first number indicates the N2C or N3C library, respectively, the letters G or m indicate a DARPin specific for GFP or mCherry, respectively, and the last two- to three-digit number is a continuous numbering of the 192 clones that were screened. 3G86.1 and 3G86.32 come from the same initial clone that turned out to be a double transformant; single transformants were obtained by plasmid extraction and retransformation.

### Protein expression and purification

The pQIq vectors containing the DARPin genes were used directly for larger scale expressions. *E. coli* XL1 blue was transformed. From a single colony an overnight culture was grown in 2YT medium with 100 µg/ml ampicillin and 1% glucose. This was used to inoculate 2YT medium containing 100 µg/ml ampicillin to an OD_600_ of 0.1 in a shake flask. This culture was grown at 37°C to an OD_600_ of 0.6–0.8 and then induced by addition of 500 µM IPTG. Five hours after induction cells were harvested by centrifugation (4000 *g*, 10 min) and resuspended in TBS_W (50 mM Tris-HCl pH 7.4, 400 mM NaCl, 20 mM imidazole, 10% glycerol). Cells were lysed by passage through a French press and cell debris was pelleted by centrifugation (20,000 *g*, 30 min). Supernatants were then applied to Ni-NTA superflow resin columns (Qiagen) and washed with 30 column volumes of TBS_W. Proteins were eluted with TBS_E (same as TBS_W but containing 250 mM imidazole).

### ELISA assay

96-well Maxisorp plates (Nunc) were coated with 100 µl NeutrAvidin (33 nM in PBS, Thermo Scientific) for 1 h and then blocked with 200 µl PBS-TB (PBS with 0.1% Tween-20 and 0.2% bovine serum albumin). After washing (three times with 300 µl PBS-T) wells were coated with 100 µl in-vivo biotinylated autofluorescent proteins (200 nM in PBS-TB), and control wells were incubated with PBS-TB only. After washing, IMAC purified DARPins were applied (100 µl, 200 nM in PBS-TB). DARPins were detected via an anti-RGS-His6 antibody (Qiagen, 1:5000 in PBS-TB), an anti-mouse-IgG conjugated to alkaline phosphatase (Pierce, 1:10,000 in PBS-TB) and p-nitrophenylphosphate (3 mM in 50 mM NaHCO_3_, 50 mM MgCl_2_). The first and second antibody was each applied for 1 h with three washing steps after incubation. After incubation with substrate for about 1 h at room temperature the absorbance at 405 nm was measured on a Tecan M1000 plate reader. All values were determined in duplicates. Note that 3m160 shows a very weak binding signal with mRuby2 in this ELISA, but this phenomenon is also seen with other DARPins, including the control DARPin off7, and thus likely an unspecific interaction with mRuby2 (Fig. 1A).

### Size exclusion chromatography (SEC)

DARPins after IMAC purification were analyzed at a concentration of 10 µM on a Superdex 75 5/150 GL column (GE Healthcare) using an Äkta Micro system (GE Healthcare) with PBS as the running buffer. Absorbance at 280 nm was recorded; for analysis the maximal absorbance value was normalized to 1 to compensate for different extinction coefficients of the DARPins. 2m22 and 2m74 which had very low extinction coefficients at 280 nm were analysed by absorbance at 230 nm. β-amylase (200 kDa), bovine serum albumin (66 kDa), carbonic anhydrase (29 kDa) and cytochrome c (12.4 kDa) were used as molecular mass standards.

### Fluorescence anisotropy assay (FA)

The whole assay was performed at room temperature in PBS (pH 7.4) containing 0.03% bovine serum albumin (BSA) (unless stated otherwise) in black non-binding 96-well plates (Greiner). Constant amounts of autofluorescent proteins (GFP, sfGFP or mCherry) at a concentration below the expected K_D_ were titrated with a dilution series of DARPins (four replicates). The mixtures were left to equilibrate for 1 h. FA was measured on a Tecan Safire II plate reader. Mean anisotropy values (a dimensionless quantity) were normalized by subtraction of the mean anisotropy value of the sample containing the lowest DARPin concentration. These normalized mean values were plotted against the DARPin concentration. The K_D_ was determined by fitting the data to a simple 1:1 binding model using SigmaPlot. For the epitope-binning assays, all measurements were carried out with DARPin concentrations high enough that all targets were completely saturated (2 µM), GFP was used at 50 nM. GFP was incubated either without any DARPin, with one DARPin or with two DARPins. Anisotropy was measured on a Tecan Safire II plate reader; all samples were prepared in duplicates.

### Surface plasmon resonance (SPR)

All SPR experiments were performed on a ProteOn XPR36 instrument on a NLC chip, using PBS containing 0.005% Tween-20 as running buffer. In-vivo biotinylated GFP was used as ligand. For kinetic measurements a kinetic titration approach (Karlsson et al., 2006) was used, since no regeneration condition could be found, that (i) removed all bound DARPins from the surface and (ii) left the immobilized GFP intact. In short, after immobilization of GFP in two ligand channels (replicates) five consecutive injections of the same DARPin with increasing concentrations were run in the same analyte channel. Data were referenced (interspot and buffer control) and injections were concatenated in the ProteOn manager software. These final datasets were then exported to a .txt-file and imported to the BiaEvaluation software, where they were fitted to a kinetic titration model described (Karlsson et al., 2006). This model also contained a term for baseline drift (which was set to 0) and a mass transfer constant k_t_, which was manually set to 10^20^, which is several orders of magnitudes above k_a_ (ca. 10^14^-fold), since there was no evidence of mass transfer.

For the epitope binning experiments, GFP was immobilized in one ligand channel, then 1 µM of one DARPin was injected in all analyte channels. A second injection was performed as soon as the instrument was ready, at which point 1 µM of each GFP binding DARPin was injected in one of the analyte channels. Data were referenced in the ProteOn manager software and inspected manually.

### Cell culture

HeLa S3 cells were cultured in DMEM GlutaMAX (GIBCO) containing 10% FBS (GIBCO). Sub-confluent cells were split 1:10 into chamber slides (Ibidi). The next day, cells were transfected with 0.2 µg of the corresponding DNA using TransIT (Mirus), according to the manufacturer's protocol. 24 hours later, cells were washed with PBS and fixed with 4% formaldehyde in PBS for 10 minutes. Cells were washed again twice with PBS and imaged using a confocal microscope, Leica SP5. Images were processed using ImageJ.

### Molecular biology

All DARPin fusion proteins were generated in pcDNA3 plasmids using the CMV promoter. Genetic constructions were carried out using restriction-free cloning (Bryksin and Matsumura, 2010) using Phusion polymerase (NEB). Primer sequences are available upon request.

### Drosophila melanogaster

Flies were grown on standard fly medium supplemented with yeast. NSlmb-3G86.32 was cloned in pUAS_attB to generate transgenic flies using the zh-86Fb landing platform (Bischof et al., 2007). Beside this strain, the following fly strains were used in this study: *en*-Gal4 (Bloomington Drosophila Stock Center (BDSC)), *tub*-Gal4 (BDSC), UAS_mCherry-NLS (Caussinus et al., 2008), ubiHis2Av::EYFP (Rebollo and González, 2004), *sqhAX3*; *sqhSqh::GFP* (Royou et al., 2004) and Gal4^332.3^ (BDSC).

### Danio rerio

Zebrafish were maintained at standard conditions (Westerfield, 1993). Fertilized eggs of wild type embryos (ABC) were injected at the 1-cell stage with 25 ng/µl of respective plasmid DNA. Plasmid h2afx:EGFP-Rab5c (Clark et al., 2011) was used to label the Rab5c vesicles. At ∼36 hpf embryos with transient expression of fluorescent markers were chosen and mounted in a 35 mm glass bottom Petri dish (0.17 mm, MatTek), using 0.7% low melting agarose (Sigma) containing 0.08% tricaine and 0.003% PTU. A Leica TCS SP5 confocal microscope with a 40× water objective was used for imaging.

## Supplementary Material

Supplementary Material
