# Supplementary material for: Protein interference applications in cellular and developmental biology using DARPins that recognize GFP and mCherry
Source: Biol Open. 2014 Nov 21;3(12):1252–61. doi: 10.1242/bio.201410041 (PMC4265764; doi:10.1242/bio.201410041)
Supplement: Supplementary Material [file supp_3_12_1252__index.html]

Protein interference applications in cellular and developmental biology using DARPins that recognize GFP and mCherry — Supplementary Material 

# Protein interference applications in cellular and developmental biology using DARPins that recognize GFP and mCherry

## bio.201410041 Supplementary Material

**Files in this Data Supplement:**

- Supplementary Material - Michael Brauchle et al. doi: 10.1242/bio.201410041
