# Supplementary material for: Protein interference applications in cellular and developmental biology using DARPins that recognize GFP and mCherry
Source: Biol Open. 2014 Nov 21;3(12):1252–61. doi: 10.1242/bio.201410041 (PMC4265764; doi:10.1242/bio.201410041)
Supplement: Supplementary Material [file supp_bio.201410041_bio.201410041-s1.pdf]

Biology Open (2014) 000, 1–10 doi:10.1242/bio.201410041

Supplementary Material

Michael Brauchle et al. doi: 10.1242/bio.201410041

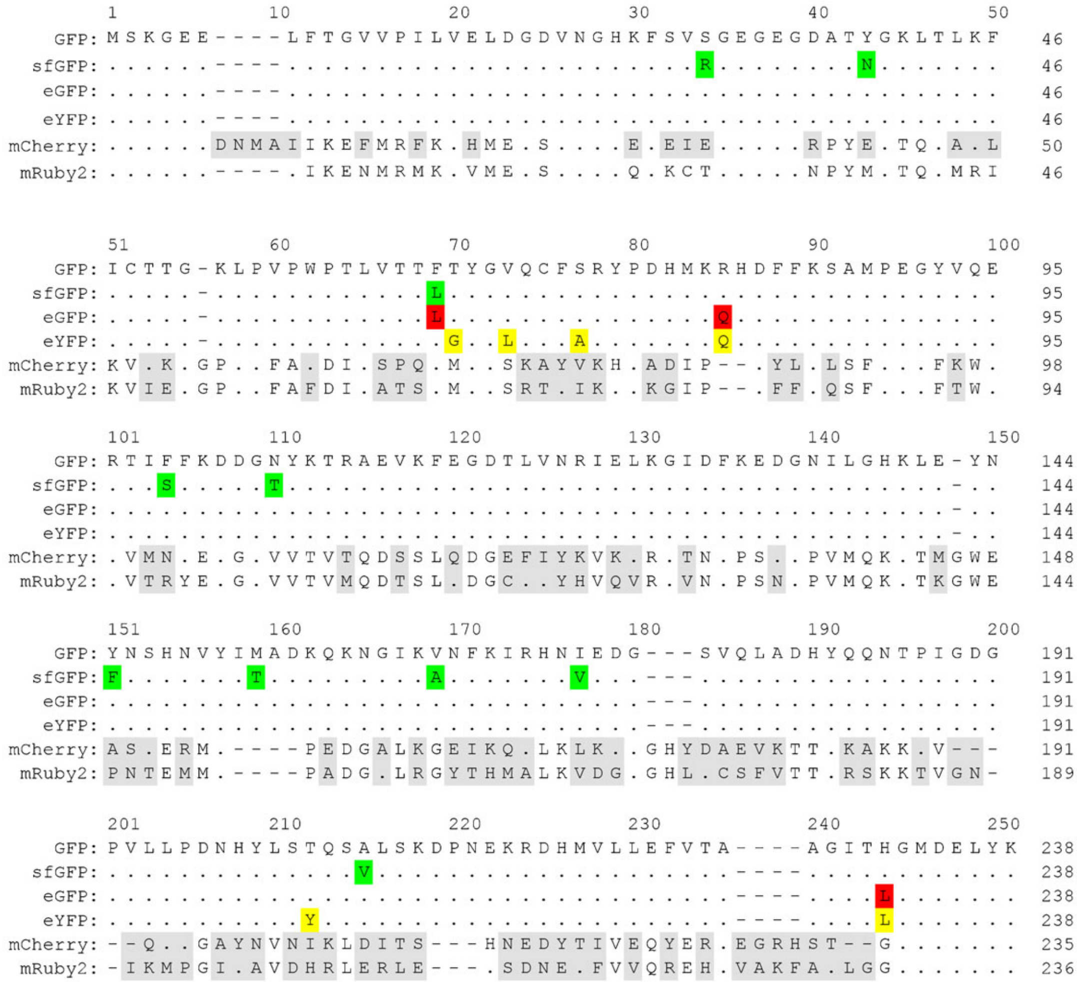

Fig. S1. Alignment of GFP, sfGFP, eGFP, eYFP, mCherry and mRuby2. Sequence-based alignment of GFP, sfGFP, eGFP, eYFP, mCherry and mRuby2. Identical residues to the GFP sequence (top line) are represented by a dot (.), gaps are shown by hyphens (-). Differences to GFP are written in one letter amino acid code and are highlighted in green, red and yellow for sfGFP, eGFP and eYFP, respectively. Differences between mCherry and mRuby2 are highlighted in gray. Compared to GFP (which was used in the RD selection) sfGFP differs in 10 residues (96% sequence identity), eGFP in 3 residues (99%) and eYFP in 6 residues (97%). mCherry (which was the target used in a different RD selection) and mRuby2 have sequence identities of 27 and 30% to GFP, respectively, and 54% to each other.

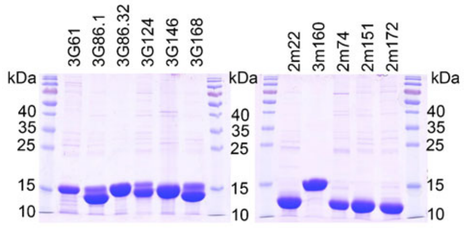

Fig. S2. Analysis of IMAC-purified DARPins. IMAC-purified DARPins were run on a 15% acrylamide gel. Note that some DARPins run as double bands; this however, is a known phenomenon: since DARPins are very stable they tend to not fully unfold despite an incubation in SDS loading buffer at 96˚C (Binz et al., 2003).

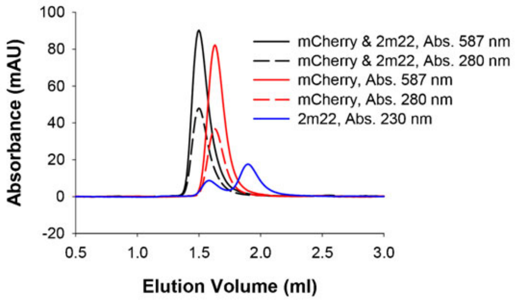

Fig. S3. 2m22 binding to mCherry analyzed by SEC. 2m22 and mCherry were run on SEC either alone or as an equimolar mixture. Elution of mCherry was followed by absorption at 280 nm and at its absorption maximum at 587 nm. Elution of 2m22 was followed by absorption at 230 nm (due to very low extinction coefficient at 280 nm). Upon co-incubation of 2m22 and mCherry a clear and complete shift towards higher molecular weights can be seen, indicating that also the dimeric fraction of 2m22 converts into a 1:1 complex with mCherry.

Biology Open (2014) 000, 1–10 doi:10.1242/bio.201410041

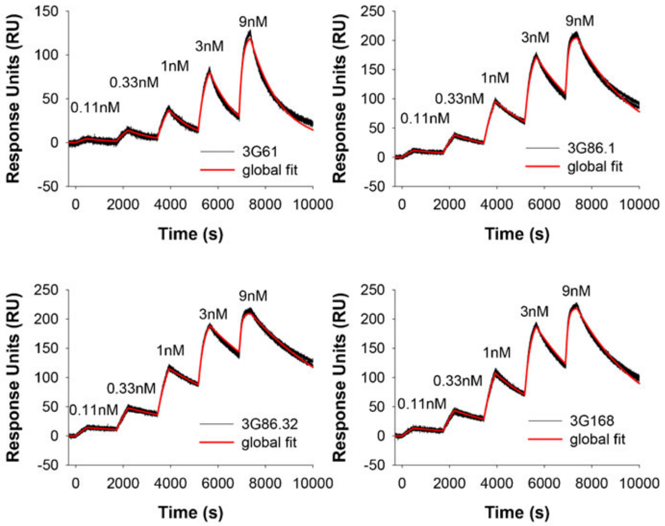

Fig. S4. Kinetic titration SPR experiments of DARPins binding to GFP. Five increasing DARPin concentrations (0.11, 0.33 1, 3, and 9 nM) were injected over a GFP-coated surface and binding was monitored by SPR. Black curves represent duplicates of binding signals, red curves indicate a global fit to a 1:1 kinetic titration binding model (Karlsson et al., 2006). Extracted kinetic data can be found in Table 1.

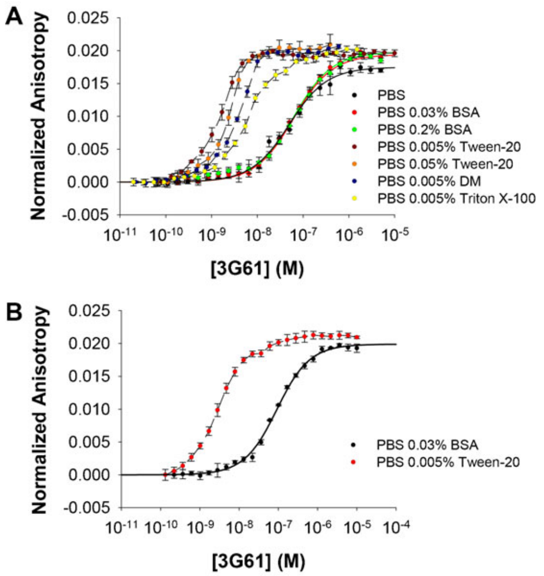

Fig. S5. Influence of buffer conditions on the affinity of anti-GFP DARPin 3G61 binding to GFP (A) and sfGFP (B). Fluorescence anisotropy assays with anti-GFP DARPin 3G61 were carried out in different buffer conditions. (A) Binding to GFP: addition of BSA does not alter the K_D as assays performed in PBS, PBS with 0.03% BSA and PBS with 0.2% BSA all give K_Ds of about 60 nM (Table 1). In contrast, addition of Tween-20, decyl-maltoside (DM) and Triton X-100 increases the affinity, although to different extents; note that the dashed lines do not represent a fitted curve, as these assay conditions are not suitable to obtain a quantitative result (as [GFP] is higher than K_D). Nonetheless, it is obvious that the addition of Tween-20 increases the affinity to around the same K_D value determined by SPR (supplementary material Fig. S4 and Table 1), measured under the same conditions. (B) Also binding to sfGFP is much tighter in PBS with Tween-20; also here the dashed line does not represent a fit. For the affinity in PBS with 0.03% BSA, see Table 1.

Biology Open (2014) 000, 1–10 doi:10.1242/bio.201410041

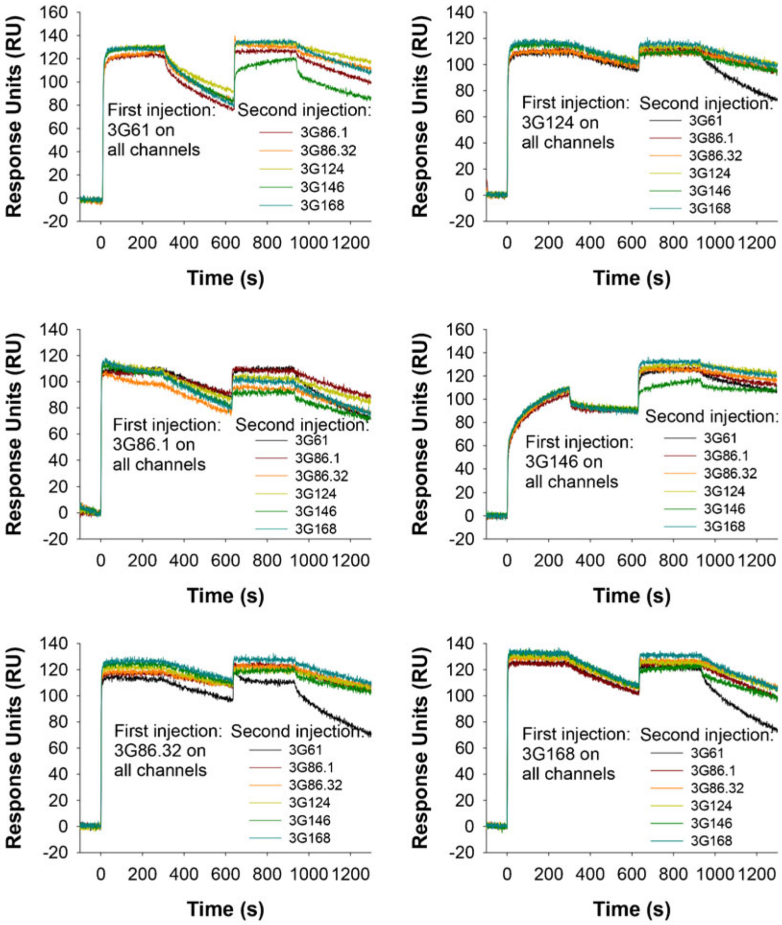

Fig. S6. SPR does not show simultaneous binding of any two DARPins to GFP. A GFP-coated SPR surface was saturated by injection of one DARPin, dissociation was allowed to occur for 300 sec, followed by a second injection with another DARPin. No combination of two anti-GFP DARPins showed an increased binding above the plateau reached by the injection of the first anti-GFP DARPin alone, indicating that these DARPins cannot bind simultaneously.

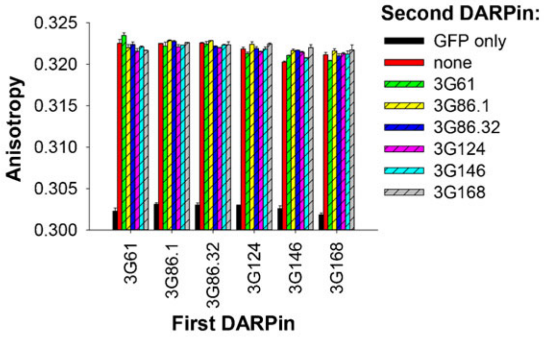

Fig. S7. Epitope binning using FA. GFP was incubated either alone, with an excess of one or with a combination of two DARPins in excess and anisotropy was measured. No combination of any two DARPins showed an increase of anisotropy above the level reached by a single DARPin, indicating that no larger complexes are formed. Bars represent mean values of two replicates, error bars represent standard deviations.

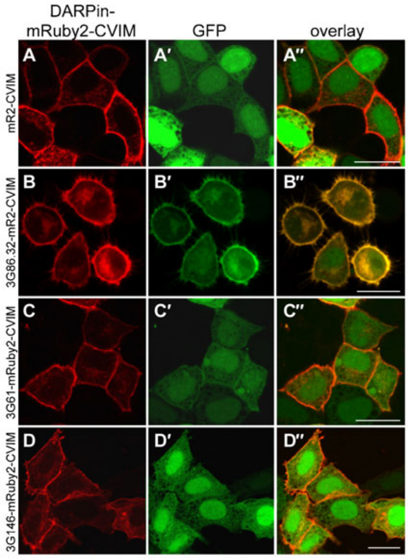

Fig. S8. Membrane-tethered anti-GFP-DARPin 3G86.32 can recruit GFP in HeLa cells. Shown are HeLa cells transiently overexpressing different DARPin-mRuby2 versions tethered to the plasma membrane (DARPin-mRuby-CVIM, A–D) together with GFP (A′–D′) to indicate whether or not GFP is recruited to the plasma membrane (A″–D″). (A,A″) mRuby2-CVIM does not recruit unfused GFP to the membrane. (B,B″) High-affinity 3G86.32-mRuby2-CVIM recruits GFP to the membrane, as can be seen in the overlap of GFP and mRuby2 signal. (C,C″) Low-affinity 3G61-mRuby2-CVIM and (D,D″) low-affinity 3G146-mRuby2-CVIM cannot re-localize GFP. Unprimed letters, mRuby2 channel; primed letters, GFP channel; double primed letters, overlay. Scale bars are 20 μm.

Biology Open (2014) 000, 1–10 doi:10.1242/bio.201410041

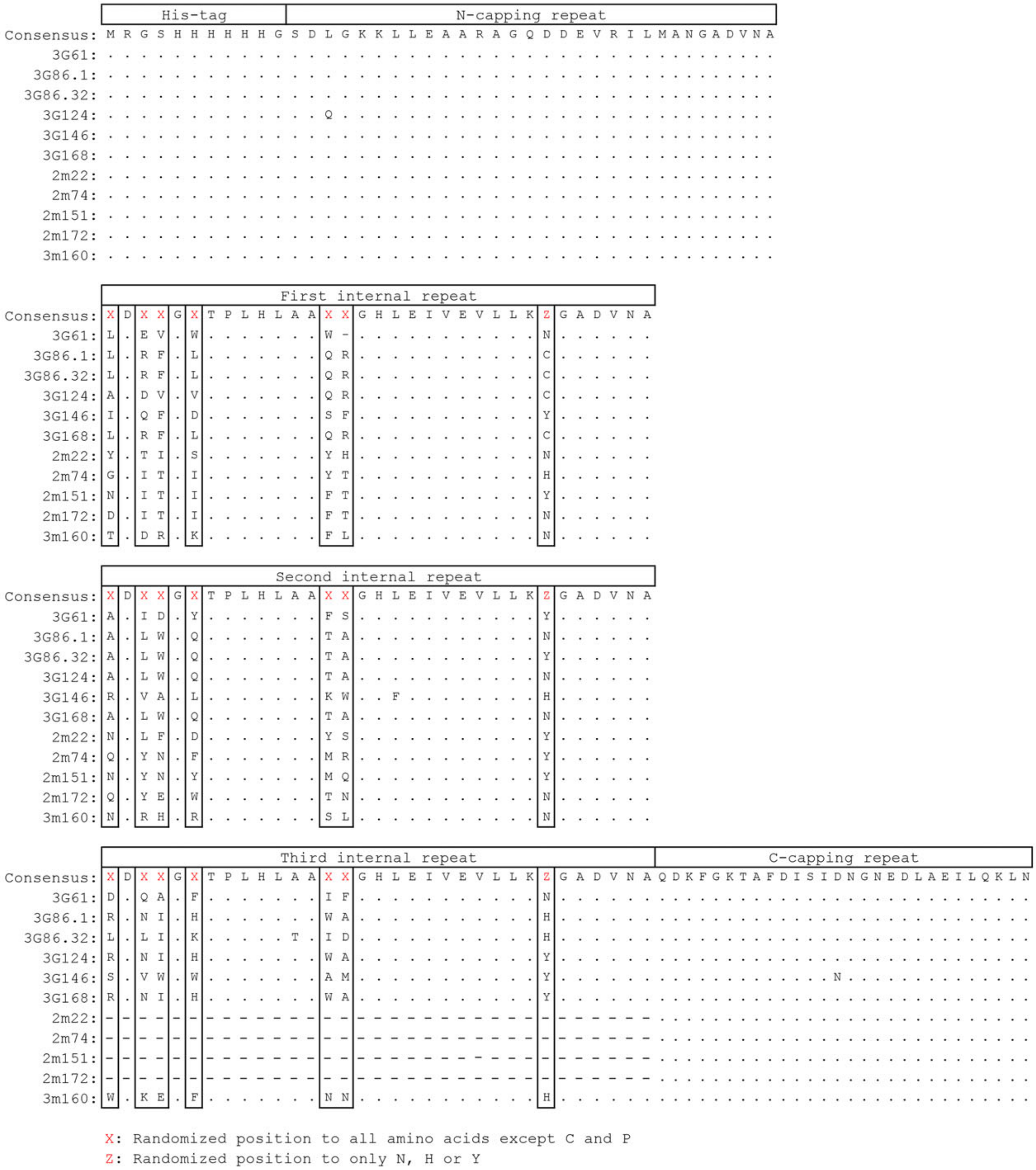

Fig. S9. Alignment of DARPins. Sequence-based alignment of selected DARPins. The top row indicates the consensus sequence used in the library with randomized positions indicated as X (randomization to all amino acids but Cys and Pro) and Z (randomization to Asn, His or Tyr) highlighted in black frames. Identical residues are shown as dots (.), gaps are shown as hyphens (-), selected randomized positions along with framework mutations are shown as one-letter amino acid code.

Biology Open

Biology Open (2014) 000, 1–10 doi:10.1242/bio.201410041

Table S1. Full-length nt sequences of DARPins used in this study

3G61:
ATGAGAGGATCGCATCACCATCACCATCACGGATCCGACCTGGGTAAGAAACTGCTGGAAGCTGCTCGTGCTGGTCAGGACGACGAAGT
TCGTATCCTGATGGCTAACGGTGCTGACGTTAACGCTCTTGACGAGGTTGGTTGGACTCCGCTGCACCTGGCTGCTTGGGGTCACCTGGA
AATCGTTGAAGTTCTGCTGAAGAACGGTGCTGACGTTAACGCTGCTGACATTGATGGTTATACTCCGCTGCACCTGGCTGCTTTTTCTGGT
CACCTGGAAATCGTTGAAGTTCTGCTGAAGTACGGTGCTGACGTTAACGCTGATGACCAGGCTGGTTTTACTCCGCTGCACCTGGCTGCT
ATTTTTGGTCACCTGGAAATCGTTGAAGTTCTGCTGAAGAACGGTGCTGACGTTAACGCTCAGGACAAATTCGGTAAGACCGCTTTCGAC
ATCTCCATCGACAACGGTAACGAGGACCTGGCTGAAATCCTGCAAAAGCTTAATTAA

3G86.1:
ATGAGAGGATCGCATCACCATCACCATCACGGATCCGACCTGGGTAAGAAACTGCTGGAAGCTGCTCGTGCTGGTCAGGACGACGAAGT
TCGTATCCTGATGGCTAACGGTGCTGACGTTAACGCTCTTGACCGTTTTGGTCTTACTCCGCTGCACCTTGCTGCTCAGCGTGGTCACCTG
GAAATCGTTGAAGTTCTGCTGAAGTGTGGTGCTGACGTTAACGCTGCTGACCTTTGGGGTCAGACTCCGCTGCACCTGGCTGCTACTGCT
GGTCACCTGGAAATCGTTGAAGTTCTGCTGAAGAACGGTGCTGACGTTAACGCTCGTGACAATATTGGTCATACTCCGCTGCACCTGGCT
GCTTGGGCTGGTCACCTGGAAATCGTTGAAGTTCTGCTGAAGCACGGTGCTGACGTTAACGCTCAGGACAAATTCGGTAAGACCGCTTTC
GACATCTCCATCGACAACGGTAACGAGGACCTGGCTGAAATCCTGCAAAAGCTTAATTAA

3G86.32:
ATGAGAGGATCGCATCACCATCACCATCACGGATCCGACCTGGGTAAGAAACTGCTGGAAGCTGCTCGTGCTGGTCAGGACGACGAAGT
TCGTATCCTGATGGCTAACGGTGCTGACGTTAACGCTCTTGACCGTTTTGGTCTTACTCCGCTGCACCTTGCTGCTCAGCGTGGTCACCTG
GAAATCGTTGAAGTTCTGCTGAAGTGTGGTGCTGACGTTAACGCTGCTGACCTTTGGGGTCAGACTCCGCTGCACCTGGCTGCTACTGCT
GGTCACCTGGAAATCGTTGAAGTTCTGCTGAAGTACGGTGCTGACGTTAACGCTCTTGACCTTATTGGTAAGACTCCACTGCACCTGACT
GCTATTGATGGTCACCTGGAAATCGTTGAAGTTCTGCTGAAGCACGGTGCTGACGTTAACGCTCAGGACAAATTCGGTAAGACCGCTTTC
GACATCTCCATCGACAATGGTAACGAGGACCTGGCTGAAATCCTGCAAAAGCTTAATTAA

3G124:
ATGAGAGGATCGCATCACCATCACCATCACGGATCCGACCAGGGTAAGAAACTGCTGGAAGCTGCTCGTGCTGGTCAGGACGACGAAG
TTCGTATCCTGATGGCTAACGGTGCTGACGTTAACGCTGCTGACGATGTTGGTGTTACTCCTCTGCACCTGGCTGCTCAGCGTGGTCACCT
GGAAATCGTTGAAGTTCTGCTGAAGTGCGGTGCTGACGTTAACGCTGCTGACCTTTGGGGTCAGACTCCGCTGCACCTGGCTGCTACTGC
TGGTCACCTGGAAATCGTTGAAGTTCTGCTGAAGAACGGTGCTGACGTTAACGCTCGTGACAATATTGGTCATACTCCGCTGCACCTGGC
TGCTTGGGCTGGTCACCTGGAAATCGTTGAAGTTCTGCTGAAGTACGGTGCTGACGTTAACGCTCAGGACAAATTCGGTAAGACCGCTTT
CGACATCTCCATCGACAACGGTAACGAGGACCTGGCTGAAATCCTGCAAAAGCTTAATTAA

3G146:
ATGAGAGGATCGCATCACCATCACCATCACGGATCCGACCTGGGTAAGAAACTGCTGGAAGCTGCTCGTGCTGGTCAGGACGACGAAGT
TCGTATCCTGATGGCTAACGGTGCTGACGTTAACGCTATTGACCAGTTTGGTGATACTCCGCTGCACCTGGCTGCTTCTTTTGGTCACCTG
GAAATCGTTGAAGTTCTGCTGAAGTACGGTGTTGACGTTAACGCTCGTGACGTTGCTGGTCTTACTCCGCTGCACTTGGCTGCTAAGTGG
GGTCACTTTGAAATCGTTGAAGTTCTGCTGAAGCACGGTGCTGACGTTAACGCTTCTGACGTTTGGGGTTGGACTCCGCTGCACCTGGCT
GCTGCTATGGGTCACCTGGAAATCGTTGAAGTTCTGCTGAAGTACGGTGCTGACGTTAACGCTCAGGACAAATTCGGTAAGACCGCTTTC
GACATCTCCATCAACAACGGTAACGAGGACCTGGCTGAAATCCTGCAAAAGCTTAATTAA

3m160:
ATGAGAGGATCGCATCACCATCACCATCACGGATCCGACCTGGGTAAGAAACTGCTGGAAGCTGCTCGTGCTGGTCAGGACGACGAAGT
TCGTATCCTGATGGCTAACGGTGCTGACGTTAACGCTACTGACGATCGTGGTAAGACTCTGCTGCACCTGGCTGCTTTTCTTGGTCACCTG
GAAATCGTTGAAGTTCTGCTGAAGAACGGTGCTGACGTTAACGCTAATGACCGTCATGGTCGTACTCCGCTGCACCTGGCTGCTTCTCTT
GGTCACCTGGAAATCGTTGAAGTTCTGCTGAAGAACGGTGCTGACGTTAACGCTTGGGACAAGGAGGGTTTTACTCCGCTGCACCTGGC
TGCTAATAATGGTCACCTGGAAATCGTTGAAGTTCTGCTGAAGCACGGTGTTGATGTTAACGCTCAGGACAAATTCGGTAAGACCGCTTT
CGACATCTCAATCGACAACGGTAACGAGGACCTGGCTGAAATCCTGCAAAAGCTTAATTAATGA

3G168:
ATGAGAGGATCGCATCACCATCACCATCACGGATCCGACCTGGGTAAGAAACTGCTGGAAGCTGCTCGTGCTGGTCAGGACGACGAAGT
TCGTATCCTGATGGCTAACGGTGCTGACGTTAACGCTCTTGACCGTTTTGGTCTTACTCCGCTGCACCTTGCTGCTCAGCGTGGTCACCTG
GAAATCGTTGAAGTTCTGCTGAAGTGTGGTGCTGACGTTAACGCTGCTGACCTTTGGGGTCAGACTCCGCTGCACCTGGCTGCTACTGCT
GGTCACCTGGAAATCGTTGAAGTTCTACTGAAGAACGGTGCTGACGTTAACGCTCGTGACAATATTGGTCATACTCCGCTGCACCTGGCT
GCTTGGGCTGGTCACCTGGAAATCGTTGAAGTTCTGCTGAAGTACGGTGCTGACGTTAACGCTCAGGACAAATTCGGTAAGACCGCTTTC
GACATCTCCATCGACAACGGTAACGAGGACCTGGCTGAAATCCTGCAAAAGCTTAATTAA

2m22:
ATGAGAGGATCGCATCACCATCACCATCACGGATCCGACCTGGGTAAGAAACTGCTGGAAGCTGCTCGTGTTGGTCAGGACGACGAAGT
TCGTATCCTGATGGCTAACGGTGCTGACGTTAACGCTTATGACACTATTGGTTCTACTCCGCTGCACCTGGCTGCTTATCATGGTCACCTG
GAAATCGTTGAAGTTCTGCTGAAGAACGGTGCTGACGTTAACGCTAATGACCTTTTTGGTGATACTCCGCTGCACCTGGCTGCTTATTCTG
GTCACCTGGAAATCGTTGAAGTTCTGCTGAAGTACGGTGCTGACGTTAACGCTCAGGACAAATTCGGTAAGACCGCTTTCGACATCTCCA
TCGACAACGGTAACGAGGACCTGGCTGAAATCCTGCAAAAGCTTAAT

2m74:
ATGAGAGGATCGCATCACCATCACCATCACGGATCCGACCTGGGTAAGAAACTGCTGGAAGCTGCTCGTGCTGGTCAGGACGACGAAGT
TCGTATCCTGATGGCTAACGGTGCTGACGTTAACGCTGGTGACATTACTGGTATTACTCCGCTGCACCTGGTTGCTTATACTGGTCACCTG
GAAATCGTTGAAGTTCTGCTGAAGCACGGTGCTGACGTTAACGCTCAGGACTATAATGGTTTTACTCCGCTGCACCTGGCTGCTATGCGT
GGTCACCTGGAAATCGTTGAAGTTCTGCTGAAGTACGGTGCTGACGTTAACGCTCAGGACAAATTCGGTAAGACCGCTTTCGACATCTCC
ATCGACAACGGTAACGAGGACCTGGCTGAAATCCTGCAAAAGCTTAATTAATGA

2m151:
ATGAGAGGATCGCATCACCATCACCATCACGGATCCGACCTGGGTAAGAAACTGCTGGAAGCTGCTCGTGCTGGTCAGGACGACGAAGT
TCGTATCCTGATGGCTAACGGTGCTGACGTTAACGCTAATGACATTACTGGTATTACTCCGCTGCACCTGGCTGCTTTTACTGGTCACCTG
GAAATCGTTGAAGTTCTGCTGAAGTACGGTGCTGACGTTAACGCTAATGACTATAATGGTTATACTCCGCTGCACCTGGCTGCTATGCAG
GGTCACCTGGAAATCGTTGAAGTTCTGCTGAAGTACGGTGCTGACGTTAACGCTCAGGACAAATTCGGTAAGACCGCTTTCGACATCTCC
ATCGACAACGGTAACGAGGACCTGGCTGAAATCCTGCAAAAGCTTAATTAATGA

2m172:
ATGAGAGGATCGCATCACCATCACCATCACGGATCCGACCTGGGTAAGAAACTGCTGGAAGCTGCTCGTGCTGGTCAGGACGACGAAGT
TCGCATCCTGATGGCTAACGGTGCTGACGTTAACGCTGATGACATTACTGGTATTACTCCGCTGCACCTGGCTGCTTTTACTGGTCACCTG
GAAATCGTTGAAGTTCTGCTGAAGAACGGTGCTGACGTTAACGCTCAGGACTATGAGGGTTGGACTCCGCTGCACCTGGCTGCTACTAA
TGGTCACCTGGAAATCGTTGAAGTTCTGCTGAAGAACGGTGCTGACGTTAACGCTCAGGACAAATTCGGTAAGACCGCTTTCGACATCTC
CATCGACAACGGTAACGAGGACCTGGCTGAAATCCTGCAAAAGCTTAATTAATGA
